# Cardiovascular and Psychiatric Morbidity in Obstructive Sleep Apnea (OSA) with Insomnia (Sleep Apnea Plus) versus Obstructive Sleep Apnea without Insomnia: A Case-Control Study from a Nationally Representative US Sample

**DOI:** 10.1371/journal.pone.0090021

**Published:** 2014-03-05

**Authors:** Madhulika A. Gupta, Katie Knapp

**Affiliations:** Department of Psychiatry, Schulich School of Medicine and Dentistry, University of Western Ontario, London, Ontario, Canada; Texas A & M, Division of Cardiology, United States of America

## Abstract

**Purpose:**

To evaluate cardiovascular and psychiatric morbidity in patient visits with obstructive sleep apnea (OSA) with insomnia (*OSA+Insomnia*) versus OSA without insomnia (*OSA-Insomnia*) in a nationally representative US sample.

**Methods:**

A retrospective case-control study of epidemiologic databases (National Ambulatory Medical Care Survey and National Hospital Ambulatory Medical Care Survey) representing an estimated ± standard error (SE) 62,253,910±5,274,747 (unweighted count = 7234) patient visits with diagnosis of OSA from 1995–2010, was conducted. An estimated 3,994,104±791,386 (unweighted count = 658) were classified as *OSA+Insomnia* and an estimated 58,259,806±4,849,800 (unweighted count = 6576) as *OSA-Insomnia*. Logistic regression analysis was carried out using *OSA+Insomnia* versus *OSA-Insomnia* as the dependent variable, and age (>50 years versus ≤50 years), sex, race (‘White’ versus ‘non-White’), essential hypertension, heart failure, ischemic heart disease, cardiac dysrhythmia, cerebrovascular disease, diabetes, obesity, hyperlipidemia, depressive, anxiety, and adjustment disorders (includes PTSD), hypersomnia and all medications used as independent variables. All comorbidities were physician diagnosed using the ICD9-CM.

**Results:**

Among patient visits with OSA, an estimated 6.4%±0.9% also had insomnia. Logistic regression analysis revealed that the *OSA+Insomnia* group was significantly more likely to have essential hypertension (all ICD9-CM codes 401) (OR = 1.83, 95% CI 1.27–2.65) and provisionally more likely to have cerebrovascular disease (ICD9-CM codes 430–438) (OR = 6.58, 95% CI 1.66–26.08). The significant OR for cerebrovascular disease was considered provisional because the unweighted count was <30.

**Conclusions:**

In a nationally representative sample, *OSA+Insomnia* was associated significantly more frequently with essential hypertension than *OSA-Insomnia*, a finding that has not been previously reported. In contrast to studies that have considered patient self-reports of psychological morbidity, the absence of a significant association with psychiatric disorders in our study may be indicative of the fact that we considered only physician-rated psychiatric syndromes meeting ICD9-CM criteria. Our findings among the *OSA+Insomnia* group are therefore most likely conservative.

## Introduction

Obstructive sleep apnea (OSA) [1] and insomnia [2], [3] are two of the most commonly encountered sleep disorders. It is well recognized that both OSA [4]–[6] and insomnia [7], [8] are independently associated with cardiovascular disease; and OSA [9], [10] and insomnia [10], [11] are also independently associated with psychiatric disorders. Activation of the hypothalamic-pituitary-adrenal (HPA) axis is believed to be one of the major mechanisms underlying the pathogenesis of both the cardiovascular and psychiatric disorders that are encountered in OSA and insomnia.

OSA is characterized by intermittent hypoxia, which can lead to oxidative stress, systemic inflammation, vascular endothelial dysfunction, and an increase in sympathetic nervous system (SNS) activity, putting OSA patients at risk for cardiovascular disorders [6], [12]. The allostatic challenge induced by the arousals of OSA is also thought to activate the HPA axis [13], [14]. This elevated SNS and HPA axis activity can lead to an upregulation of the renin-angiotensin-aldosterone system, potentially leading to the development of hypertension [13], [15]. Elevated SNS and HPA activity can also lead to an increase in cortisol levels, which have been implicated in psychiatric disorders such as depression [16], [17]. Various studies have also associated insomnia with activation of the HPA axis and SNS [18]–[21]. Corticotropin releasing factor hyperactivity, due to early-life stress or genetic predisposition, can lead to an amplification of the stress response, followed by prolonged and exaggerated sleep difficulties following stress and the subsequent development of chronic insomnia [20], [22]. This chronic hyperarousal puts insomnia patients at risk for cardiovascular disease [23] and mood disorders [20] also associated with HPA axis hyperactivity. Insomnia may be a consequence of the enhanced HPA axis activity seen in OSA [13], [14], [18]. Alternatively, insomnia may cause or exacerbate OSA by creating repeated sleep fragmentation, which can cause disruptions of the upper airway muscle tone, leading to greater airway collapsibility and the development of OSA [18], [24], [25]. The shared physiological mechanism of HPA hyperactivity underlying both OSA and insomnia may explain the high comorbidity of these two sleep disorders.

The comorbidity of OSA and insomnia, also referred to as ‘Sleep apnea plus’ [26], was reported over 4 decades ago [27] and various studies [26], [28]–[42] have examined the prevalence of insomnia in sleep apnea reporting prevalence rates ranging from 7.3% [34] to 57.6% [28]. To our knowledge, there are 3 reported studies [28], [34], [36] that have directly [36] or indirectly [28], [34] examined the frequency of both cardiovascular and psychiatric comorbidities in OSA with insomnia in epidemiologically representative samples. There are differences in the definition of the primary mediating variable of insomnia in each of the studies [34], [36]. A study of data from the 2005–2008 United States National Health and Nutrition Examination Survey (NHANES) [36] which largely uses patient-reports of health professional diagnosed disorders, reports a 43% frequency of insomnia in OSA (n = 546) versus 30% frequency of insomnia in individuals without OSA (n = 12,047). In this study [36] the participants were asked questions regarding the frequency of difficulty falling asleep, of prolonged nocturnal awakenings, and of undesired early morning awakening over the past month. Individuals who described experiencing at least one of any of the above insomnia symptoms ‘often’ (defined as 5–15 times per month) or ‘almost always’ (defined as 16–30 times per month) were classified as having insomnia. Results from the Icelandic Sleep Apnea Cohort Study [28] indicate a 57.6% frequency of insomnia in polysomnographically diagnosed obstructive sleep apnea (n = 824) versus 31% among controls from the general population (n = 762) in Iceland. Insomnia was defined [28] using answers to 2 questions from the Basic Nordic Sleep Questionnaire which described the patients' experience over the past month: ‘I have difficulties falling asleep at night’ and ‘I wake up often during the night’. The patients rated their response on a 5-point scale where a rating of ‘i’ denoted ‘never/almost never’, a rating of ‘ii’ denoted ‘less than once a week’, a rating of ‘iii’ denoted ‘once or twice a week’, a rating of ‘iv’ denoted ‘three to five times a week’, a rating of ‘v’ denoted ‘every day or almost every day of the week’. Those with scores ≥4 were defined as having insomnia. Finally, data from the prospective Norwegian Hordaland Health Study (n = 6892) found a much lower frequency of comorbid insomnia in sleep apnea of 7.3% versus a 4.9% frequency of insomnia without sleep apnea [34]. In this study [34], more stringent diagnostic criteria were used including an assessment of the effect of insomnia on functioning and longer duration of symptoms, and this may explain the lower frequency of comorbid sleep apnea and insomnia in this sample. Insomnia symptoms were assessed [34] using the Karolinska Sleep Questionnaire where insomnia diagnosis was based upon 4 items each rated along a 5-point scale with ratings denoting ‘never’, ‘rarely (a few times per year)’, ‘sometimes (a few times per month)’, ‘mostly (several times a week)’, or ‘always’. Subjects were categorized as having insomnia if they reported sleep onset insomnia, sleep maintenance insomnia, early morning wakening or a combination, ‘several times a week’ or ‘always’ during the past 3 months, in addition to reporting impaired work performance during the preceding year caused by sleep problems.

The role of comorbid insomnia on OSA severity remains inconclusive as insomnia complaints in OSA have been associated with more severe OSA [35], [43], milder OSA [31] and no significant objective differences in sleep parameters [26], [28], [37], [44] in various studies. The co-occurrence of OSA and insomnia has other potentially important clinical implications as it could have an additive effect on the comorbidities that have been independently associated with both of these conditions. Studies that have examined comorbidities in OSA with insomnia generally report a higher frequency of emotional and psychiatric symptoms [26], [28], [31], [34]–[36], [44], [45], but not cardiovascular symptoms including patient-reported physician diagnosed hypertension [28] and patient-reported health-professional diagnosed hypertension, myocardial infarction, congestive heart failure and stroke [36]. One exception to these findings is the recent report by Sivertsen et al. [34] that found a significantly higher frequency of patient-reported stroke, but not angina or myocardial infarction, in the OSA with insomnia group versus OSA alone. This divergent finding may be due to the more stringent criteria for the diagnosis of insomnia used in this study [34].

As a follow-up to recent epidemiologic studies that have directly [36] or indirectly [28], [34] addressed both cardiovascular and psychological/psychiatric morbidity in OSA plus insomnia, we carried out a more expanded analysis of several major cardiovascular and psychiatric disorders that have been previously associated with OSA alone, in our OSA plus insomnia sample. In this study we examined the frequency of physician diagnosed cardiovascular and psychiatric disorders that are commonly associated with OSA alone, among patient visits with OSA plus insomnia, in a randomly selected, nationally representative sample of patient visits in the US, to doctors' offices, and hospital outpatient and emergency departments for all possible diagnoses. In order to examine the possible effect of insomnia on OSA related comorbidities, we used patient visits with OSA without comorbid insomnia as the reference group in our analyses. The objective of this study was to evaluate the possible additive effect of comorbid insomnia in OSA on cardiovascular and psychiatric disorders that are commonly known to be associated with OSA alone, in a large nationally representative sample of patient visits for OSA, after controlling for potential confounding factors such as sex, age, race, obesity, diabetes, hyperlipidemia, all medications and hypersomnia.

## Methods

Using a retrospective cross-sectional design, we examined data collected from 1995 to 2010 by the National Ambulatory Medical Care Survey (NAMCS) and National Hospital Ambulatory Medical Care Survey (NHAMCS) [46]. The NAMCS and NHAMCS are publicly accessible databases available at http://www.cdc.gov/nchs/ahcd.htm. The University of Western Ontario Office of Research Ethics determined that ethics approval was not required for research involving publicly accessible information such as the NAMCS/NHAMCS databases. The NAMCS and NHAMCS are national surveys conducted by the National Center for Health Statistics (NCHS) at the Centers for Disease Control and Prevention (CDC), and use a multi-stage probability design to collect nationally representative samples of health care visits to physicians' offices (NAMCS) and hospital outpatient departments and emergency rooms (NHAMCS) in the US. Full details of the multi-stage data collection method are available on the NAMCS/NHAMCS website [46]. The NAMCS and NHAMCS Public Use Data files from 1995 to 2010 were merged into one database for our analyses. The NAMCS and NHAMCS represent patient visits for all possible diagnostic groups, and document up to 3 ‘reasons for visit’ and 3 diagnoses per patient visit which are diagnosed and coded by a physician using the International Classification of Diseases, 9^th^ edition, Clinical Modification (ICD9-CM) [47]. In order to preserve the confidentiality of participating patients and physicians, all survey data in the databases are masked through the inclusion of variables for stratification, sampling unit, and patient visits [46]. NCHS standards for reliability state that estimates based on fewer than 30 cases are considered unreliable [48].

### Variables studied

The following variables were created for our analyses.

1. *Sleep Apnea*: included any patient visit where one of the 3 possible diagnoses were coded using ICD9-CM code 327.23 denoting ‘Obstructive Sleep Apnea’ (first introduced in 2006), code 780.57 denoting ‘Other and Unspecified Sleep Apnea’, and code 780.53 denoting ‘Hypersomnia with Sleep Apnea’, or any patient visit where one of the 3 ‘reasons for visit’ was coded as ‘Sleep Apnea’.
2. *Insomnia*: included any patient visit where at least one of the 3 possible diagnoses was coded using ICD9-CM code 307.41 denoting ‘Transient Disorder of Initiating or Maintaining Sleep’, code 307.42 denoting ‘Persistent Disorder of Initiating or Maintaining Sleep’, code 780.51 denoting ‘Insomnia with Sleep Apnea’ or code 780.52 denoting ‘Other Insomnia’, or any patient visit where one of the 3 ‘reasons for visit’ was coded as ‘Insomnia’.
3. *Sleep Apnea plus Insomnia* (*OSA+Insomnia*): denoted patient visits with both sleep apnea and insomnia derived using the codes for the ‘*Sleep Apnea*’ and ‘*Insomnia*’ variables above.
4. *Sleep Apnea without Insomnia* (*OSA-Insomnia*): denoted patient visits with sleep apnea without insomnia, where both variables were derived using the codes for the ‘*Sleep Apnea*’ and ‘*Insomnia*’ variables above.
5. *Sleep Apnea Plus*: A dichotomous variable denoting *OSA+Insomnia* versus *OSA-Insomnia*, derived using the ‘*Sleep Apnea*’ and ‘*Insomnia*’ variables above.
6. *Cardiovascular Disorders*: The following cardiovascular disorders were coded largely based upon the literature on the cardiovascular comorbidities in OSA: (i) *Essential hypertension*: coded for patient visits with all ICD9-CM codes 401 (401, 401.0 to 401.9); (ii) *Heart failure*: coded for patient visits with all ICD9-CM codes 428 (428, 428.0 to 428.9) (iii) *Ischemic heart disease*: coded for patient visits with all ICD9-CM codes 410–414; (iv) *Cardiac dysrhythmias*: coded for patient visits with all ICD9-CM codes 427 (427, 427.0 to 427.9); and (v) *Cerebrovascular disease*: coded for all patient visits with ICD9-CM codes 430–438.
7. *Psychiatric Disorders*: The following psychiatric disorders were coded largely based upon the literature on psychiatric comorbidities in OSA: (i) *Depressive disorders*: coded for all patient visits with all (including 5^th^ digits) ICD9-CM codes 296.2 denoting ‘Major Depressive Disorder, Single Episode’, all ICD9-CM codes 296.3 denoting ‘Major Depressive Disorder, Recurrent Episode’, ICD9-CM code 296.82 denoting ‘Atypical Depressive Disorder’, and ICD9-CM code 311 denoting ‘Depressive Disorder, Not Elsewhere Classified’; (ii) *Anxiety disorders*: coded for all patient visits with all (including 5^th^ digits) ICD9-CM codes 300.0 denoting ‘Anxiety states’, ‘Anxiety state, unspecified’, ‘Panic disorder without agoraphobia’, ‘Generalized anxiety disorder’, and ‘Other anxiety states’. (iii) *Adjustment Disorders*: coded for patient visits with all (including 5^th^ digits) ICD9-CM codes 309 denoting ‘Adjustment reaction’, ‘Adjustment disorder with depressed mood’, ‘Prolonged depressive reaction’, ‘Adjustment reaction with predominant disturbance of other emotions’, ‘Adjustment disorder with disturbance of conduct’, ‘Adjustment disorder with mixed disturbance of emotions and conduct’, ‘Other specified adjustment reactions’ including ‘Posttraumatic stress disorder’, and ‘Unspecified adjustment reaction’.
8. *Confounding factors*: The following conditions that are commonly encountered in both cardiovascular and psychiatric disorders were controlled for statistically: (i) *Hyperlipidemia*: included any patient visit where at least one of the 3 possible diagnoses were coded using ICD9-CM codes 272.0 to 272.4, which denote hypercholesterolemia, hyperglyceridemia, hyperlipidemia, and hyperchylomicronemia; (ii) *Obesity*: included any patient visit where at least one of the 3 possible diagnoses were coded using the ICD9-CM codes 278, 278.0, 278.00, 278.01 related to obesity and morbid obesity; (iii) *Diabetes Mellitus*: included any patient visit where at least one of the 3 possible diagnoses were coded using ICD9-CM codes 250, which code for both non-insulin dependent and insulin- dependent diabetes mellitus and their complications; (iv) *Medications*: denoted patient visits associated with use of 1 to 8 medications (in NAMCS/NHAMCS from 1995–2002, one to six medications per visit were coded, from 2003 to 2010 one to eight medications per visit were coded) versus patient visits with no medications. This variable was created as a wide range of medications can theoretically confound the association between sleep apnea and insomnia. A separate variable ‘*Z drugs*’ was also created to just denote zaleplon, zolpidem, and eszopiclone. The ‘Z drugs’ variable was created to test whether hypnotic medication use in our sample of *OSA+Insomnia* patients was consistent with earlier reports [26], [33] of increased hypnotic use in *OSA+Insomnia* patients, however the more general ‘*Medications*’ variable was used to control statistically for the possible confounding effect of any medications that the patients might have been using. A ‘*Tobacco smoking*’ variable was also created to denote patient visits associated with tobacco smoking versus all other visits (including visits coded as non-smokers, non-responders or ‘blank’ or unknown in the various studies). There was some lack of consistency in the way the patient visits not associated with tobacco smoking were coded in the databases from 1995 to 2010.
9. *Demographic factors*: (i) *Age* was categorized as >50 years versus ≤50 years; (ii) *Race* was categorized as a dichotomous variable denoting ‘White’ and ‘Non-White’ (which included ‘Black’, ‘Asian’, ‘Native Hawaiian’, ‘Pacific Islander’, ‘American Indian’, ‘Alaska Native’, and ‘More than one race reported’). Age, race and sex were controlled for in our statistical analyses.
10. *Hypersomnia*: included any patient visit where at least one of the 3 possible diagnoses were coded using ICD9-CM code 780.54 denoting ‘Other hypersomnia’, or any patient visit where one of the 3 ‘reasons for visit’ was coded as ‘Hypersomnia’. We controlled for hypersomnia as it can confound the type of insomnia the patient is experiencing. Our study sample did not delineate the type of insomnia eg., sleep onset, sleep maintenance or early morning wakening, and sleep maintenance insomnia in OSA has been associated with hypersomnia [28]. Secondly, insomnia and hypersomnia can coexist in many cases of OSA [31], and the presence of hypersomnia in the *OSA+Insomnia* patient could be a confounding factor for other comorbidities.

### Statistical analysis

All analyses were conducted using the Complex Samples module of SPSS version 20 [49] in order to account for the multistage probability sampling design used by the NAMCS and NHAMCS. The relation of the *Sleep Apnea Plus* variable (ie., *OSA+Insomnia* versus *OSA-Insomnia*) to the cardiovascular and psychiatric disorders was examined using logistic regression analysis using *Sleep Apnea Plus* as the dependent variable, after controlling for potential confounders such as age (>50 years versus ≤50 years), sex (male vs female), race (‘White’ versus ‘non-White’), ‘*Diabetes mellitus*’, ‘*Obesity*’, ‘*Hyperlipidemia*’, ‘*Hypersomnia’* and ‘*Medications*’ use. The smoking variable was not used in the logistic regression model as there was no significant difference in the frequency of smoking among the *OSA+Insomnia* versus *OSA-Insomnia* groups. Odds ratios (OR) were calculated to determine whether the frequencies of the independent variables were significantly different between the *OSA+Insomnia* versus *OSA-Insomnia* groups.

## Results

The frequency of *OSA+Insomnia* in our sample of sleep apnea patient visits (total unweighted count = 7234) was 6.4%±SE (±0.9%) (6.1%±0.9% male and 6.9%±1.2% female) (unweighted count = 658). Table 1 summarizes the demographic characteristics of the sample. Compared to *OSA-Insomnia*, patients with *OSA+Insomnia* were older (p<0.05) and more likely (p<0.001) to be prescribed hypnotics such as the ‘*Z drugs*’ (zolpidem, eszopiclone, zaleplon). Table 2 summarizes the results of the logistic regression analysis using ‘*Sleep Apnea Plus*’ (*OSA+Insomnia* vs *OSA-Insomnia*) as the dependent variable. The logistic regression model indicates that the frequency of patients over age 50 years is no longer significantly higher (Odds ratio or OR = 1.23, 95% CI 0.87–1.74) in the *OSA+Insomnia* group versus the *OSA-Insomnia* group, after controlling for comorbidities, other demographic and potential confounding factors. In contrast to the *OSA-Insomnia* group, there was a lower frequency of ‘*Obesity*’ (OR = 0.08, 95% CI 0.03–0.22) in the *OSA+Insomnia* group. As for comorbidities, in contrast to the *OSA-Insomnia* group, the *OSA+Insomnia* group had a significantly higher frequency of ‘*Essential hypertension*’ (OR = 1.83, 95% CI 1.27–2.65) and ‘*Cerebrovascular disease*’ (OR = 6.58, 95% CI 1.66–26.08). Among the *OSA+Insomnia* group, an estimated 14.5% (±1.3%) had ‘*Essential hypertension*’ (total unweighted count in OSA sample = 523; unweighted count = 35 in *OSA+Insomnia* group) in contrast to 9.4% (±0.6%) in the *OSA-Insomnia* group. Similarly, among the *OSA+Insomnia* group, an estimated 1.6% (±0.9%) had a diagnosis of ‘*Cerebrovascular disease*’ (total unweighted count in OSA sample = 24, unweighted count = 4 in *OSA+Insomnia* group) in contrast to 0.4% (±0.2%) in the *OSA-Insomnia* group. The cerebrovascular disease finding is provisional as the unweighted count was <30. There were no significant differences (Table 2) in the frequencies of the other cardiovascular diagnostic groups including ‘*Heart failure*’, ‘*Cardiac dysrhythmia*’, and ‘*Ischemic heart disease*’, or psychiatric disorders including ‘*Depressive disorders*’, ‘*Anxiety disorders*’, or *‘Adjustment Disorders’*. However, it should be noted that these groups all had unweighted counts of <30. Similarly, sex, race, and potential confounders including ‘*Diabetes*’, ‘*Hyperlipidemia*’, ‘*Medication use*’ and ‘*Hypersomnia*’ did not differ between the *OSA+Insomnia* versus *OSA-Insomnia* groups.

**Table 1:** Demographic and clinical features of the obstructive sleep apnea (OSA) sample.

Demographic or Clinical Feature	All OSA visits	*OSA+Insomnia*	*OSA-Insomnia*
Estimated (±SE) number of visits (unweighted count)	62,253,910±5,274,747 (7234)	3,994,104±791,386 (658)	58,259,806±4,849,800 (6576)
Age* (±SE) years	50.80±0.59	53.35±1.13	50.62±0.61
Sex (±SE) % female (F) vs male(M)	39.5%±1.3% F, 60.5%±1.3% M	42.6%±3.9% F, 57.4%±3.9% M	60.7%±1.3%F, 39.3%±1.3%M
Race (±SE) %: ‘White’(W) vs%‘Black/African American (B)’ vs %‘Other’^a^	86.1%±0.9% W, 10.1%±0.8% B, 3.8%±0.5% ‘Other’	85.6%±3.7% W, 11.4%±3.1% B, 3.1%±2.2% ‘Other’	86.1%±0.9% W, 10.1%±0.8% B, 3.8%±0.4% ‘Other’
‘Z drugs’* (zolpidem, eszopiclone, zaleplon) use %(±SE)	3.7%±0.6%	12.4%±1.9%	3.1%±0.6%
Tobacco Smoking% (±SE): ‘Smokers’ (S) vs All other visits ‘Other’ (O)^b^	11.1%±0.8% S, 88.9%±0.8% O	8.3%±2.0 S, 91.7%±2.0 O	11.3%±0.8 S, 88.7%±0.8 O

* Significantly (p<0.05) different between the *OSA+Insomnia* vs. *OSA-Insomnia* groups.

a ‘Other’ denotes: Asian/Native Hawaiian/Pacific Islander/American Indian/Alaska Native/More than one race reported.

b ‘Other’ responses denotes: non-smoker, blank, and unknown.

**Table 2:** Results of logistic regression analysis using *Sleep Apnea Plus* as the dependent variable.

Independent Variable	Wald Statistic (Wald F)	Significance level (Bonferroni Correction)	Odds ratio (95% CI) (*OSA+Insomnia* vs *OSA-Insomnia*)*
‘*Essential hypertension*’	10.400	0.001	1.83 (1.27–2.65)
‘*Cerebrovascular disease*’	7.209	0.007	6.58 (1.66–26.08)
‘*Heart failure*’	0.937	0.333	0.42 (0.07–2.46)
‘*Cardiac dysrhythmia*’	0.007	0.934	0.93 (0.15–5.90)
‘*Ischemic heart disease*’	0.896	0.344	0.39 (0.05–2.77)
‘*Depressive disorders*’	0.129	0.720	0.81 (0.26–2.51)
‘*Anxiety disorders*’	0.000	0.988	1.01 (0.27–3.75)
*‘Adjustment Disorders’*	0.704	0.402	0.40 (0.05–3.38)
Sex (male vs. female)	0.408	0.523	0.89 (0.62–1.28)
Race (White vs. Non-White)	0.090	0.764	0.91 (0.47–1.74)
‘*Obesity*’	25.434	0.000	0.08 (0.03–0.22)
‘*Diabetes*’	0.887	0.347	0.55 (0.16–1.90)
‘*Hyperlipidemia*’	0.010	0.921	1.07 (0.25–4.68)
‘*Medications*’ (any medication vs no medication)	3.833	0.051	0.63 (0.39–1.00)
‘*Hypersomnia*’	0.888	0.346	1.47 (0.66–3.29)

*Corrected model using all variables significant at p<.001, with pseudo R^2^ (Nagelkerke's) = 0.046.

## Discussion

We examined the frequency and comorbidity of *OSA+Insomnia* with cardiovascular and psychiatric disorders that are commonly associated with obstructive sleep apnea alone, in a nationally representative sample of an estimated (±SE) 62,253,910±5,274,747 (unweighted count = 7234) patient visits for obstructive sleep apnea in the US, from 1995 to 2010. To our knowledge, our study represents the largest sample of patients with *OSA+Insomnia* that have been examined to date [36]. In order to examine the possible effect of insomnia on OSA related comorbidities, we used patient visits with OSA without comorbid insomnia (*OSA-Insomnia*) as the reference group in our analyses. The estimated (±SE) frequency of insomnia in our obstructive sleep apnea sample was 6.4%±SE (±0.9%), which is lower than the 21.9% to 57.6% prevalence of insomnia in sleep apnea reported in many previous studies [26], [28]–[33], [35], [36] and comparable to the 7.3% prevalence of insomnia in obstructive sleep apnea reported in a recent epidemiologic study [34], which used more stringent criteria to diagnose insomnia. The wide range of reported frequencies most likely are a reflection of the wide range of criteria used to diagnose insomnia, most of which do not use the effect of insomnia on daytime functioning in their diagnostic criteria. In our study, insomnia was diagnosed if one of the 3 possible diagnoses for the patient visit was coded by the physician using the ICD9-CM codes (307.41, 307.42, 780.51, 780.52) for insomnia or if one of the 3 possible ‘reasons for visit’ for the patient was coded by the physician as ‘insomnia’, suggesting that the symptom of insomnia would have to be significant and likely severe for a patient to report insomnia as one of their chief complaints during a visit to their doctor's office or hospital, and our results therefore are likely to be conservative. The relatively lower frequency of *OSA+Insomnia* in our sample of OSA patient visits therefore most likely also is a reflection of the more conservative criteria used to diagnose insomnia in our study, and consistent with other studies [34] that have employed more stringent criteria to diagnose insomnia.

As for other possible confounding factors, the frequency of ‘*Obesity*’ was significantly lower (OR = 0.08, 95% CI 0.03–0.22) in the *OSA+Insomnia* group, which is similar to the findings of one study [36]; other studies have found no difference [28] and higher body mass index [34] in the *OSA+Insomnia* group. ‘*Diabetes*’ and ‘*Hyperlipidemia*’ were not significantly different between *OSA+Insomnia* versus *OSA-Insomnia*, which is consistent with previous studies that have examined diabetes [28], [34], [36] and total cholesterol [36] in *OSA+Insomnia*. ‘*Hypersomnia*’ was not significantly different between our *OSA+Insomnia* group versus *OSA-Insomnia*. This is in contrast to the report that OSA patients with a disorder of maintaining sleep were sleepier, as measured by the Epworth Sleepiness Scale, than OSA patients without a disorder of maintaining sleep [28]. Since we did not have information about whether patients had a disorder of initiating or maintaining sleep, our non-significant findings most likely represent the possible heterogeneity of the insomnia diagnosis in our sample. As expected, the *OSA+Insomnia* group was more likely to be using hypnotic medications (Table 1); however, overall, there was no significant difference in the frequency of overall medication use (Table 2) between *OSA+Insomnia* and *OSA-Insomnia*.

There was a significantly higher frequency of ‘*Essential hypertension*’ (OR = 1.83, 95% CI 1.27–2.65) and ‘*Cerebrovascular disease*’ (OR = 6.58, 95% CI 1.66–26.08) in the *OSA+Insomnia* versus the *OSA-Insomnia* group, suggesting an additive effect of insomnia on these disorders. This has not been previously reported. Essential hypertension and cerebrovascular disease were both physician diagnosed at the time of the study and coded using the ICD9-CM [47], and this finding among a randomly selected epidemiologically representative sample of patient visits most likely represents a clinically significant finding. The NCHS standards for reliability require that estimates be based on at least 30 sample records [48]; our findings related to ‘*Essential hypertension*’ (unweighted cases in *OSA+Insomnia* = 35) are therefore definitive, while findings related to ‘*Cerebrovascular disease*’ (unweighted cases in *OSA+Insomnia* = 4) should be considered provisional, because of the smaller number of unweighted cases. Previous studies have not found a higher frequency of hypertension [28], [34], [36] in *OSA+Insomnia*, where patients were categorized as having hypertension using the following criteria: if they had indicated that they had been diagnosed by a doctor and were on antihypertensive medication [28]; if they self-reported health-professional diagnosed hypertension at any point and had 3 consecutive blood pressure measurements where systolic blood pressure was >140 mm Hg and diastolic blood pressure was >90 mm Hg [36]; or had higher systolic blood pressure at baseline [34]. As for cerebrovascular disease, one study [34] reported a higher frequency of strokes in *OSA+Insomnia* and the patient was coded as having a stroke based upon a positive response to the question whether they have had a stroke, and the diagnostic criteria for insomnia were the most stringent. While another study [36], which used less stringent diagnostic criteria for insomnia, and in which a patient was coded as having a stroke based upon self-reported health-professional diagnosis of stroke at any time, did not find a positive association between stroke and *OSA+Insomnia*. Similar to our study, previous studies [34], [36] did not find a significantly (p<0.05) higher frequency of angina or myocardial infarction [34] and myocardial infarction or congestive heart failure [36] in their *OSA+Insomnia* sample. We also examined the frequency of ICD9- CM coded cardiac dysrhythmias, as arrythmias such as atrial fibrillation have been associated with OSA, and may be a feature of ANS activation, however we found no significant differences in their frequency in the *OSA+Insomnia* sample in comparison to the *OSA-Insomnia* sample.

In contrast to the results of previous studies [26], [31], [34]–[36], [44] that have compared *OSA+Insomnia* patients to those with *OSA-Insomnia*, we did not find a higher frequency of ‘*Depressive disorders*’ or ‘*Anxiety disorders*’ diagnosed using ICD9-CM criteria. One potential reason for this is the method used for assessing psychiatric/mental health symptoms. Previous studies have assessed psychiatric disorder status using established self-report scales [34], [35], [44], questionnaires designed to enquire about mood [36], mental symptoms, and previous psychiatric diagnoses [26], [31]. Those scoring high on these psychiatric scales and self-report questionnaires that usually screen for psychiatric disorders, may not have actually met all the diagnostic criteria for a psychiatric disorder. While previous studies that have employed patient-self reports of psychiatric comorbidity have shown that patients with *OSA+Insomnia* may be more likely to feel anxious or depressed [26], [34]–[36], [44], our findings indicate that those with *OSA+Insomnia* are not more likely to actually be diagnosed with a depressive or anxiety *disorder* than patients with *OSA-Insomnia*, diagnosed using ICD9-CM diagnostic criteria. Some studies have not noted higher frequency of psychiatric comorbidity in OSA patients with insomnia. For example, one study has reported that insomnia in patients with OSA had negative effects on depression and anxiety, but only in men, and not women [37]. Another recent study found no significant difference between those with OSA and insomnia and those with OSA alone on the Beck Depression Inventory or the State-Trait Anxiety Inventory [50]. However, they did find that those with OSA and insomnia were more likely to report having received a psychiatric diagnosis [50]. These findings suggest that there is some variability in the reports of a higher frequency of psychiatric morbidity in OSA patients with insomnia. Our sample was selected from a nationally representative sample for all possible diagnoses, and the unweighted counts of patient visits with both OSA+Insomnia and a psychiatric diagnosis was <30, making the estimate not as reliable [48], and increasing the risk of a Type II error.

Some of the limitations of this study are the fact that our results are based upon retrospective cross-sectional data and therefore it is not possible to arrive at any conclusions regarding causation. The retrospective design prevented the independent evaluation of the patients studied and checking of the diagnostic criteria used. The basic sampling unit in the NAMCS and NHAMCS is the patient visit, and it is possible that some of the data points represent the same patient seen for more than 1 visit during the data collection period. We do not have polysomnographic data to confirm the physician diagnoses of obstructive sleep apnea. While such confirmation would be ideal, it is rare for large epidemiological studies to collect such information [34], [36]. In fact, only one epidemiological study examining comorbidities in *OSA+Insomnia* used polysomnography to make an OSA diagnosis [28]. Other epidemiological studies have used less stringent criteria than the present study to diagnose OSA including self-report of physician diagnosis [36] and two self-report questions on snoring and breathing cessation during sleep [34]. As such, our results represent relatively stringent diagnostic criteria for OSA.

Another potential confounder in our database is the fact that the ICD9- CM diagnostic codes used to denote sleep apnea (ICD9-CM codes 780.53 denoting ‘Hypersomnia with Sleep Apnea’ and 780.57 denoting ‘Other and Unspecified Sleep Apnea’) did not specifically mention obstructive sleep apnea, until the ICD9-CM code 327.23 denoting ‘Obstructive Sleep Apnea’ was first introduced in 2006. However, the earlier codes alone have been used to diagnose obstructive sleep apnea in other reports from our database [51]. We also diagnosed obstructive sleep apnea when one of the 3 possible ‘reasons for visit’ was coded as ‘sleep apnea’. We excluded patient visits coded as Cheyne-Stokes respiration and central sleep apnea (added to ICD9-CM from 2006 onwards) from our OSA variable, and therefore it is unlikely that we have inadvertently included central sleep apnea cases. The above factors could have led to the misclassification of some patient visits as obstructive sleep apnea, however this is mitigated by the fact that the comparison group also consisted of patients with sleep apnea (without insomnia), which would potentially cancel out some of the error introduced by the diagnostic codes used to code for obstructive sleep apnea. Finally, the unweighted number of cases of cerebrovascular disease which is significantly more frequent in *OSA+Insomnia* is <30 and therefore the estimate does not meet the criteria for reliability [48] and the findings related to cerebrovascular disease provide directions for future studies and can only be considered to be provisional.

## Conclusions

Our findings that hypertension and possibly cerebrovascular disease are more common in those with *OSA+Insomnia* are consistent with theories suggesting that a possible linking mechanism between insomnia and OSA is the hyperactivity of the HPA axis [14], [18], [30] and the sympathetic nervous system [18]. OSA leads to HPA hyperactivity, which then leads to hyperarousal and insomnia, which perpetuates these enhanced cortisol levels, and eventually leads to the development of other diseases associated with HPA hyperactivity like cardiovascular disease [13], [14]. If this theory is correct, then once the OSA has been addressed, one would expect to also see improvements in insomnia, and cardiovascular symptoms. Studies have shown that once OSA is addressed with continuous positive airway pressure (CPAP), surgical intervention, or nasal dilator strip therapy, insomnia symptoms [52]–[55] and symptoms of hypertension [56] improve dramatically. Having insomnia actually also predicts poorer CPAP adherence [40], [57]. These findings stress the important association between insomnia and sleep apnea, and highlight the necessity of correct diagnosis of comorbid *OSA+Insomnia*.
